# Meta-analysis of promoter methylation in eight tumor-suppressor genes and its association with the risk of thyroid cancer

**DOI:** 10.1371/journal.pone.0184892

**Published:** 2017-09-19

**Authors:** Fatemeh Khatami, Bagher Larijani, Ramin Heshmat, Abbasali Keshtkar, Mahsa Mohammadamoli, Ladan Teimoori-Toolabi, Shirzad Nasiri, Seyed Mohammad Tavangar

**Affiliations:** 1 Chronic Diseases Research Center, Endocrinology and Metabolism Population Sciences Institute, Tehran University of Medical Sciences, Tehran, Iran; 2 Endocrinology and Metabolism Research Center, Endocrinology and Metabolism Clinical Sciences Institute, Tehran University of Medical Sciences, Tehran, Iran; 3 Department of Health Sciences Education Development, School of Public Health, Tehran University of Medical Sciences, Tehran, Iran; 4 Molecular Medicine Department, Pasteur Institute of Iran, Tehran, Iran; 5 Department of Surgery, Tehran University of Medical Sciences, Shariati Hospital, Tehran, Iran; 6 Department of Pathology, Dr. Shariati Hospital, Tehran University of Medical Sciences, Tehran, Iran; Hokkaido Daigaku, JAPAN

## Abstract

Promoter methylation in a number of tumor-suppressor genes (TSGs) can play crucial roles in the development of thyroid carcinogenesis. The focus of the current meta-analysis was to determine the impact of promoter methylation of eight selected candidate TSGs on thyroid cancer and to identify the most important molecules in this carcinogenesis pathway. A comprehensive search was performed using Pub Med, Scopus, and ISI Web of Knowledge databases, and eligible studies were included. The methodological quality of the included studies was evaluated according to the Newcastle Ottawa scale table and pooled odds ratios (ORs); 95% confidence intervals (CIs) were used to estimate the strength of the associations with Stata 12.0 software. Egger’s and Begg’s tests were applied to detect publication bias, in addition to the “Metatrim” method. A total of 55 articles were selected, and 135 genes with altered promoter methylation were found. Finally, we included eight TSGs that were found in more than four studies (*RASSF1*, *TSHR*, *PTEN*, *SLC5A*, *DAPK*, *P16*, *RARβ2*, and *CDH1*). The order of the pooled ORs for these eight TSGs from more to less significant was *CDH1* (OR = 6.73), *SLC5* (OR = 6.15), *RASSF1* (OR = 4.16), *PTEN* (OR = 3.61), *DAPK* (OR = 3.51), *P16* (OR = 3.31), *TSHR* (OR = 2.93), and *RARβ2* (OR = 1.50). Analyses of publication bias and sensitivity confirmed that there was very little bias. Thus, our findings showed that *CDH1* and *SCL5A8* genes were associated with the risk of thyroid tumor genesis.

## Introduction

Thyroid cancer is the most common endocrine malignancy, and the incidence of thyroid cancer is increasing rapidly worldwide [1, 2]. Thyroid cancer includes several histological types and subtypes with various cellular origins and characteristics [3, 4] but is usually composed of two types of endocrine thyroid cells, i.e., follicular thyroid cells and para follicular C cells. The majority of thyroid malignancies, including papillary thyroid cancer (PTC), follicular thyroid cancer (FTC), poorly differentiated thyroid cancer (PDTC), and anaplastic thyroid cancer (ATC), are derived from follicular thyroid cells[2, 4]. PTC and FTC are further classified into the differentiated thyroid cancer (DTC) group [5, 6].

Recently, scientists have demonstrated the involvement of genetic and epigenetic alterations in the development and progression of thyroid cancer. In addition to common gene mutations, such as mutations in *BRAF* [7–11], matrix metalloproteinase-2 [12], Ras family genes [13, 14], phosphatase and tensin homolog (*PTEN*) [15, 16], phosphatidylinositol 3-kinase (*PIK3CA*) [17, 18], anaplastic lymphoma kinase (*ALK*) [19], β-catenin 1 (*CTNNB1*) [20, 21], isocitrate dehydrogenase 1 (*IDH1*) [22], survivin [23], and epidermal growth factor receptor (*EGFR*) [24, 25], epigenetic alterations have also been shown to be important in thyroid cancer. Alterations in the genome by means of DNA methylation or histonemodification without altering the underlying DNA sequence, resulting in changes in the expression of target genes, are called epigenetic changes [26]. The most substantial epigenetic effects are observed by aberrant gene methylation, an epigenetic hallmark of human cancers, including thyroid cancer; methylation usually silences the gene when present in the promoter regions [27]. DNA methylation is a process in which methyl groups are added to the DNA molecule, altering the activity of the DNA segment without changing the nucleotide sequence. Several reports have shown that *BRAF* mutations are associated with hypermethylation of some TSGs, such as tissue inhibitor of metalloproteinases 3 (*TIMP3*), Ras association domain family member 1 (*RASSF1*), *SLC5A*, thyroid-stimulating hormone receptor (*TSHR*), death-associated proteinkinase1 (*DAPK1*), cyclin-dependent kinase inhibitor 2A (*P16*), and retinoic acid receptor-β (*RARβ*) [28–32]. Hypo methylation and over-expression of may genes are observed in various cancers [33].

In this study, we carried out a comprehensive meta-analysis of candidate genes associated with methylation in patients with thyroid cancer. Our results provide insights into the most effective TSGs, for which the promoter methylation has been considered a risk factor of progression towards thyroid carcinogenesis.

## Materials and methods

The current meta-analysis was designed according to the latest version of the PRISMA checklist for meta-analysis guidelines S2 and S3 Figs.

### Publication selection

This study (Prospero code: CRD42016033484) was conducted using PubMed, Scopus, and Web of Science search engines. Studies published between January 1, 2000 and October 1, 2016 were considered S1 Fig. The following key words were used: “methylation” or “hyper methylation” and “thyroid cancer” or “thyroid neoplasm” or “thyroid tumor” or “thyroid carcinoma” S4 Fig. Additionally, the references of the selected articles and related review articles were manually reviewed in order to identify any additional studies.

### Inclusion and exclusion criteria

All nominated studies were reviewed by two authors independently. Studies that met our defined inclusion criteria were considered eligible for the meta-analysis. The purpose of our investigation was to identify definite gene promoter methylation in tumor tissue (fresh-frozen tissue, formalin-fixed paraffin-embedded [FFPE] samples, and plasma) from patients with thyroid cancer and normal sex- and age-matched controls. Normal controls were defined as normal adjacent tissue of thyroid cancer (NPTC), goiter, and nodular goiter (NG) samples. For normal controls, samples were the same or similar tissue type as those collected from patients with thyroid cancer. Methylation detection was based on methylation-specific polymerase chain reaction (MSP), quantitative MSP (QMSP), and combined bisulfite restriction analysis (COBRA) and methylation-specific multiplex ligation-dependent probe amplification (MS-MLPA) assays. All articles were published in English. Studies with insufficient data despite contacting the author were excluded.

### Data collection

The following information was extracted from each study: first author, year of publication, country of research, type of sample, method for methylation determination, pathological stage, type of tissue (tumor and control), name of targeted gene(s), and frequency of promoter methylation in the target gene(s) in both tumor and normal thyroid tissues.

### Quality assessment of individual studies

The quality of each study was assessed separately by two authors according to the Newcastle-Ottawa Scale (NOS) assessment tool [34]. Articles containing case control studies were scored according to the selection, comparability, and exposure. The assessment was made by scoring with stars ranging from zero to nine. Articles that scored six or more stars were qualified for inclusion into the meta-analysis.

### Statistical analysis

Stata 12.0 (Stata Corporation, TX, and USA) was used in our meta-analysis. The odds ratios (ORs) and 95% confidence intervals (CIs) were applied to evaluate the association between promoter methylation of the eight TSGs and the risk of thyroid cancer [35]. Q-tests based on the χ^2^ and I^2^ statistics were used to investigate the heterogeneity among the studies [36–38]. If substantial heterogeneity existed (*P*<0.05 for the Q statistic or I^2^>50%), a random effect model was applied to pool the ORs; if not, a fixed effect model was applied [39, 40]. In addition, a meta-regression analysis was performed to discover the underlying reasons for statistical heterogeneity. Furthermore, subgroup analysis was conducted to determine the source of the heterogeneity. Sensitivity analysis was carried out to measure the effects of single studies on the overall estimate by ignoring one study at a time. A funnel plot, the trim-and-fill method, Begg’s test, and Egger’s test were applied to assess publication bias. All tests were two-sided, and results with *P* values of less than 0.05 were considered significant.

## Results

### Study selection and characteristics

**Fig 1:**
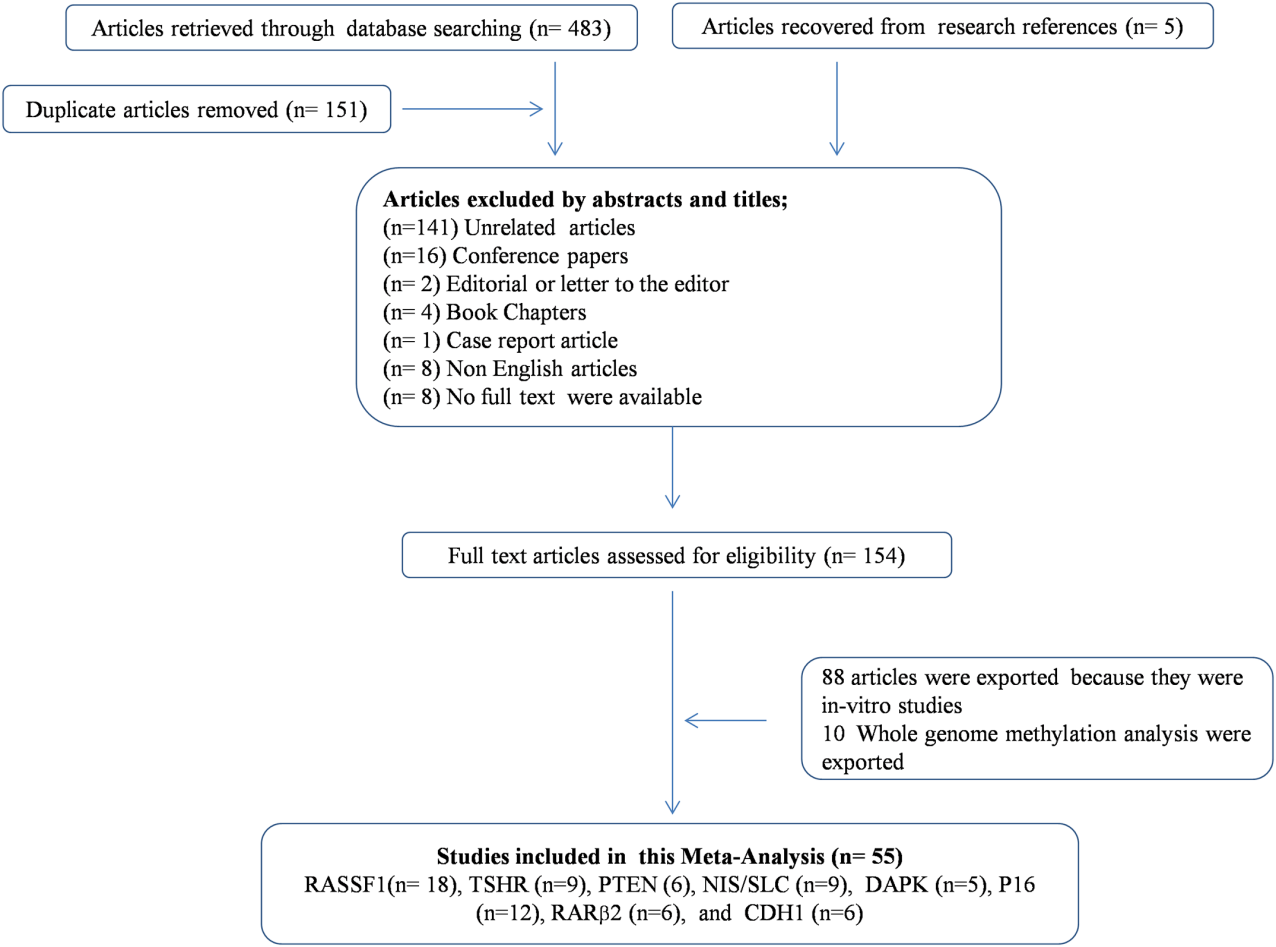
Flow diagram of study selection for the current meta-analysis.

The strategies and results of study selection are presented in Fig 1. A total of 180 articles were excluded, and the remaining 154 related articles were analyzed further. Of these, 66 articles were case/control studies, and the remaining 88 articles were in- = vitro studies. Finally, 55 studies were chosen to evaluate promoter methylation and thyroid cancer because 10 studies were based on whole-genome methylation analysis. These 55 articles evaluated a total of 135 genes, including eight genes (*RASSF1*, *TSHR*, *PTEN*, electro genic sodium and chloride-dependent sodium-coupled solute transporter [*SLC/NIS*], *DAPK*, *P16*, *RARβ2*, and cadherin 1 [*CDH1*/E-cadherin] that have been shown to be associated with thyroid cancer in more than four studies; accordingly, we selected these eight TSGs for the meta-analysis. The frequency of promoter methylation in these eight TSGs was evaluated 55 studies; 18 studies evaluated *RASSF1* (855 cases versus 379 controls), 12 studies evaluated *P16* (626 cases versus 268 controls), nine studies evaluated *TSHR* (501 cases versus 258 controls), nine studies evaluated *SLC/NIS* (340 cases versus 201 controls), six studies evaluated *PTEN* (398 cases versus 189 controls), six studies evaluated *RARβ2* (400 cases versus 132 controls), six studies evaluated *CDH1* (355 cases versus 120 controls), and five studies evaluated *DAPK* (354 cases versus 117 controls) S1 Tables.

### Meta-analysis

**Fig 2:**
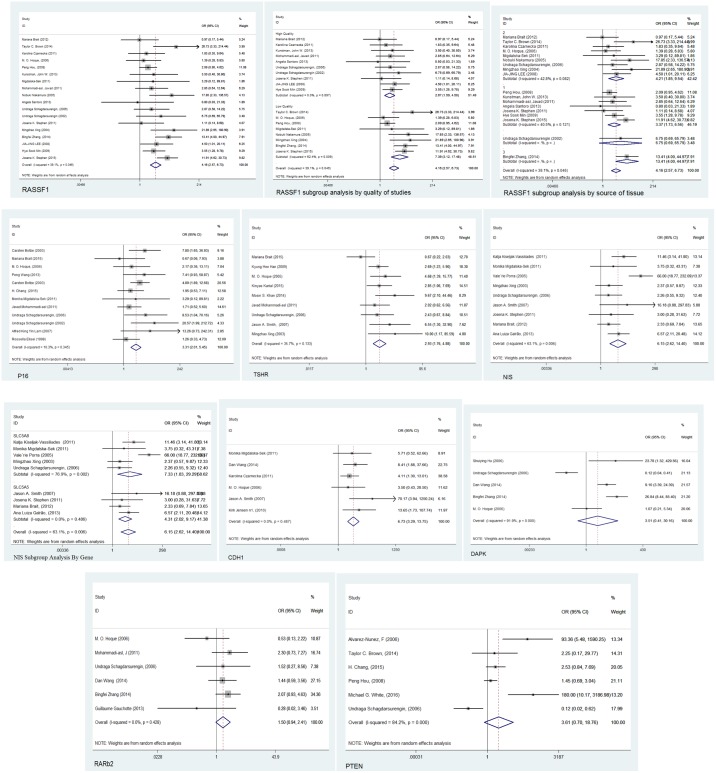
The result of meta-analysis (random-effects model. Forest plot for evaluating the association between promoter methylation in the eight tumor-suppressor genes (*RASSF1*, *P16*, *TSHR*, *PTEN*, *NIS/SLC*, *DAPK*, *RARβ2*, and *CDH1*) and thyroid cancer risk. For *RASSF1* and *NIS/SLC*, subgroup analyses are also presented. The random-effect model was used for all analyses.

Our results revealed that the frequency of promoter methylation of all eight TSGs increased in patients with thyroid cancer compared with that in controls under the random-effects model. Meta-analysis of the association between NIS hyper methylation and thyroid cancer, among 340 cases of thyroid cancer and 201 controls indicated a statistically significant difference (overall OR: 6.15, 95% CI: 2.62–14.40, *p* = 0.006). Subgroup analyses of NIS by the exact gene name indicated an OR of 7.33 for *SLC5A8* and 4.31 for *SLC5A5*. Similar results were observed for the other seven genes, including *RASSF1* (overall OR: 4.16, 95% CI: 2.57–6.73, *p* = 0.046), *TSHR* (overall OR: 2.93, 95% CI: 1.76–4.88, *p* = 0.133), *P16* (overall OR: 3.31, 95% CI: 2.01–5.45, *p* = 0.345), *PTEN* (overall OR: 3.61, 95% CI: 0.70–18.76, *p*<0.001), *DAPK* (overall OR: 3.51, 95% CI: 0.41–30.16, *p*<0.001), *CDH1*(overall OR: 6.73, 95% CI: 3.29–13.75, *p* = 0.482), and *RARβ2* (overall OR: 1.50, 95% CI: 0.94–2.41, *p*< 0.428), as shown in Fig 2. Subgroup analysis according to NOS quality grade for *RASSF1* illustrated that studies with a quality of more than 7 yielded more specific thyroid cancer risk factors (OR: 2.67) in comparison with low-quality studies (OR: 7.39). Furthermore, subgroup meta-analyses of *RASSF1* on the basis of the sampling method and tissue type showed that fresh-frozen tissues and paraffin-fixed embedded tissues were similar (OR: 4.32 and 3.37, respectively), in contrast to one study on blood samples (overall OR: 13.41, 95% CI: 4.00–44.97).

### Publication bias and sensitivity analysis

**Fig 3:**
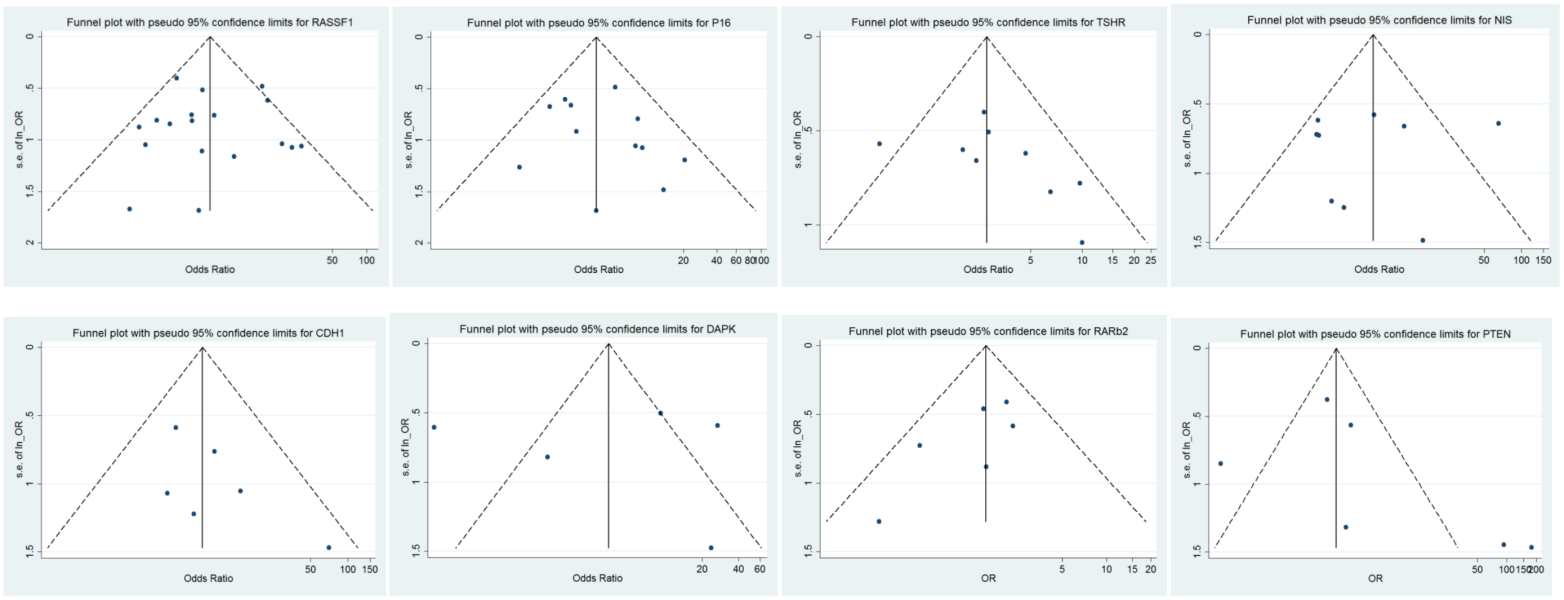
Funnel plot of publication bias. Funnel plot for evaluating the association of promoter methylation of eight tumor-suppressor genes with thyroid cancer risk.

The publication bias of each individual TGS was evaluated separately using funnel plots, the trim-and-fill method, Begg’s linear regression test, and Egger’s linear regression. For *RASSF1* and *P16* genes (n > 10 reports each), funnel plots, Begg’s linear regression tests, and the trim-and-fill method were applied for analysis publication bias. However, for the other remaining six TSGs, funnel plots, Egger’s linear regression tests, and the trim-and-fill method were used for publication bias assessment because these TSGs were reported in less than 10 studies (Fig 3).

**Fig 4:**
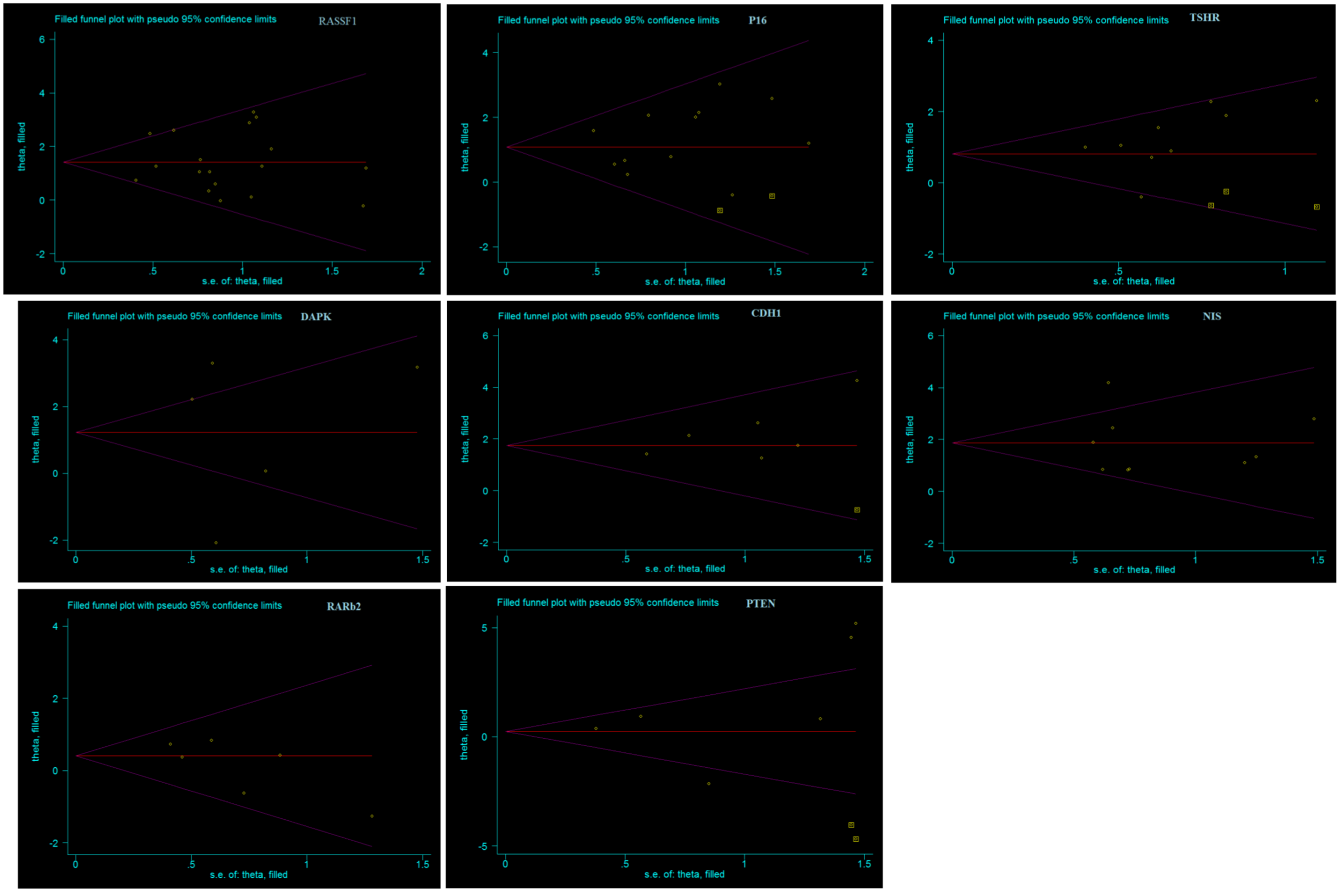
The result of Sensitivity analysis by the trim-and-fill method. Funnel graph of the trim-and-fill method for evaluating publication bias and sensitivity for eight tumor-suppressor genes.

The results of Begg’s test and Egger’s test supported that no significant publication bias existed in our analysis of the association between promoter methylation in eight TSGs and thyroid cancer risk. Begg’s test results for *RASSF1* (z = 0.08, *p* = 0.940) and *P16* (z = 0.75, *p* = 0.451) and Egger’s graphs for *TSHR*, *PTEN*, *CDH1*, *DAPK*, *SLC*, and *RARβ2* showed minimum bias. Sensitivity analysis by the trim-and-fill method specified that the results were stable in this meta-analysis, and removal of each study had no significant effect on the pooled ORs (Fig 4).

## Discussion

In mammalian cells, epigenetic alternations play important role in regulation of gene expression. As the most common epigenetic alteration, DNA methylation usually occurs in CpG island regions of the gene promoter, causing activation or inactivation of gene function. The hypermethylation of TSG promoter suppresses genes expression by inhibiting transcription, thereby affecting cell signaling pathways. Two previous meta-analyses demonstrated the association between *RASSF1A* promoter methylation and PTC risk [31, 41]. In the current report, a comprehensive review was carried out to determine the relationship between thyroid cancer susceptibility and gene methylation. Among the eight TSGs identified in our study, hyper methylation in the promoter region of seven thyroid cancer-associated genes (*RASSF1*, *TSHR*, *PTEN*, *SLC*5A, *DAPK*, *P16*, and *CDH1*) was associated with an increased risk of thyroid cancer with an OR of 2 or more, and one gene (*RARβ2*) was associated with increased risk of thyroid cancer with an OR of 2 or less.

The *CDH1* gene is a protein-coding gene that encodes a trans membrane glycoprotein localized in the adherents junctions of epithelial cells [42]. *CDH1* gene inactivation by promoter methylation in cancer contributes to the increase in the proliferation, invasion, and metastasis of tumor cells [43–50]. For this gene, the pooled OR from six studies was 6.73, and only very minor publication bias and small study effects (p = 172) were observed, consistent with previous reports [44, 50, 51].

SLCs are a collection of membrane transport proteins comprising over 400 members [52, 53]. NIS member 5 (SLC5A5), also known as sodium/iodide co transporter or solute carrier family 5, is a protein encoded by the *SLC5A5* gene in humans [54, 55]. This trans membrane glycoprotein (87 kDa and 13 trans membrane domains) transports two sodium cations (Na+) for each iodide anion (I–) into the cell [55]. In thyroid tissue, *SLC5A5* plays a crucial role in thyroid hormone (TH) biosynthesis and uptake [56–58]. Our data showed that SLS5A5 was also identified as a risk factor (OR: 6.15). SLC5A is widely expressed in human tissues, including thyroid tissue [59–61] and is frequently methylated in human cancers [33–36], including thyroid cancer [61–64]. The SLC5A Meta bias test and resulting Egger graph showed that there was no the publication bias for association between the methylation of this gene and thyroid cancer. *SLC5A8* as an another variant of SLC5 gene, transports iodide through a passive mechanism [65] and mono carboxylates short-chain fatty acids through a sodium-coupled mechanism [66]. SLC5A8 is a sodium-coupled mono carboxylate transporter; methylation of the *SLC5A8* gene is essential in human cancers [67], and the protein encoded by this gene shows structural features of a sodium-iodide symporter with 48% homology to SLC5A5 (610 amino acids) [66]. We observed significant results for *SLC5A5* and *SLC5A8* in subgroup analysis using the exact *SLC* gene name. *SLC5A8* showed the highest OR (7.33); therefore, *SLC5A8* rather than *CDH1* may be the most important gene showing hyper methylation in thyroid carcinogenesis.

Inactivation of the TSG *RASSF1* has been reported in several studies [68]. RASSF1 protein contains a Ras-association domain and can control both the cell cycle and apoptosis pathways [69–71]. In some studies, *RASSF1* promoter inactivation has been detected in more than 30% of thyroid tumors, representing the most and frequent event in thyroid cancers [72, 73]; however, in some reports, no significant correlation between *RASSF1* methylation and thyroid cancer risk was detected [68, 74–78]. In two prior meta-analyses, researchers only examined the association between *RASSF1* promoter methylation and PTC risk [31, 41]. Although the results of these prior meta-analyses demonstrated that the frequency of *RASSF1* promoter methylation was significantly associated with increased risk of thyroid cancer, our study showed that the influence of *RASSF1* promoter methylation was lower than those of *CDH1* and *SLC5A8* promoter methylation. Moreover, subgroup analysis demonstrated that *RASSF1* was a risk factor with a higher OR in low-quality studies (OR: 7.39 versus 2.67). Additionally, subgroup analysis of the sampling source demonstrated major differences among blood samples, paraffin-fixed embedded tissues, and fresh-frozen tissues (OR: 5.65 versus 1.41 and 1.81). A significant association was also found for *RASSF1* in both FTC and PTC [31]. The number of articles included in our meta-analysis of *RASSF1* was 18 when conference presentations were excluded, whereas 12 studies, including two conference papers, were included in the previous study [31]. Thus, we could conclude that promoter methylation of *RASSF1* was a risk factor for thyroid carcinogenesis, but that this gene was not as important as was previously thought. In contrast to our prediction, *RASSF1* promoter hyper methylation was not the most important candidate for thyroid cancer association because higher ORs were observed for *CDH1* and *SLC5*. The trim-and-fill method, which was not performed for *RASSF1*, implies that there was only very minor publication bias. Subgroup meta-analyses of *RASSF1* according to the type of tissue showed that the fresh-frozen tissue and paraffin-fixed embedded tissue were similar, in contrast to blood samples. Due to the limited number of blood studies (only one), our investigation did not support the use of plasma or serum as the most reliable sample source for methylation analysis [79, 80].

The *PTEN* gene, encoding a phosphatase enzyme found in virtually all tissues, is located on chromosome 10q23.3 and has a major impact on the phosphatidylinositol 3-kinase (PI3K)/AKT pathway. In fact, PTEN functions to block this signaling pathway by converting PIP3 into PIP2, thereby regulating cell proliferation and differentiation [81, 82]. PTEN point mutations, deletions, and promoter methylation have been reported in thyroid carcinoma [83–87]. Our results showed that the pooled OR was 3.61, confirming the findings of previous studies. Funnel plots with the pseudo value of the 95% CI for *PTEN*, which covered the zero point in the Egger graph, detected no publication bias. Moreover, visual inspection of the funnel plot indicated no asymmetry, suggested that there was no publication bias in the evaluation of *PTEN* methylation and thyroid cancer risk. Moreover, Egger’s test did not provide statistical evidence of the asymmetry. However, application of the trim-and-fill method and addition of two studies caused the estimated OR to be similar to the original estimate, indicating the reliability and strength of our analyses.

DAPK is a large 160-kDa protein composed of various functional domains that interacts with cytoskeleton-associated serine/threonine kinase [88–90]. The tumor-suppressor function of the *DAPK* gene was distinguished, and methylation-mediated silencing was verified in many human cancers [91]. Our findings of the pooled OR of *DAPK* methylation and its association with thyroid cancer risk supported the involvement of this gene in thyroid tumor genesis, consistent with previous reports [29, 44, 46, 49, 85, 92]. A Meta bias study with Egger’s test showed that there was no small study effect (p = 976) and no publication bias.

The *P16* (*CDKN2a*/*INK4a*) gene is an important TSG that is involved in the P16/cyclin-dependent kinase/retinoblastoma pathway and acts as a negative regulator of the cell cycle [93, 94]. The results of our pooled OR for *P16*, with the exclusion of Meta bias (which showed no publication bias), demonstrated that *P16* was a thyroid cancer risk factor, consistent with previous studies [87, 95, 96], with the exception of one study showing opposite results [74].

The *TSHR* gene, located on chromosome 14q31, is a G protein-coupled receptor that regulates signaling across the cell membrane of follicular cells in the thyroid tissue. After activation by TSH, TSHR generates corresponding effects in the cell using second messengers. Deregulation of TSHR plays a crucial role in thyroid carcinogenesis [97–99]. Hyper methylation of the *TSHR* promoter is frequently found in thyroid carcinoma, although the promoter is un-methylated in normal and benign thyroid tumors [28, 85, 98–100]. The pooled OR in our study indicated that *TSHR* had an important role in thyroid cancer. Using Egger’s test results and the trim-and-fill method, we found that there was no publication bias.

In PTC, several methylation studies have shown that *RARβ* promoter methylation is significantly altered [28, 46, 101]. Quantitative assessments of *RARβ* and its association with *BRAF* mutations have revealed promoter methylation of this gene in thyroid tumor genesis [28, 29, 85, 92, 102]. *RARβ* has been shown to be associated with thyroid cancer recurrence; however, in our meta-analysis, it was not possible to examine this concept by subgroup analyses due to the low sample size and lack of data related to recurrence in the identified studies. Our results for pooled ORs highlighted the accuracy of the previous studies. Funnel plots with pseudo 95% CIs for *RARβ2* and covering the zero point in Egger graphs detected no publication bias. Moreover, visual inspection of the funnel plots showed no asymmetry; therefore, there was no publication bias in our evaluation of *PTEN* methylation and thyroid cancer risk. Egger’s test also did not display statistical evidence of asymmetry (*P* = 0.115). The trim-and-fill method was applied to check the precision of our results.

In this meta-analysis, minimum publication bias was detected in the qualified studies for all eight TSGs. Begg’s test, Egger’s test, and funnel plots indicated that the data did not show a significant discrepancy among all studies. Furthermore, consistent results were found in the sensitivity analysis. However, there were several limitations to this analysis, including the influence of the small sample size in cases and controls and the non achievable cut-off point of methylation.

## Conclusion

Taken together, our findings showed that promoter methylation of the *CDH1* gene played an important role in thyroid cancer initiation and progression. Promoter methylation in this gene was found to be a promising biomarker for the early diagnosis of thyroid cancer. Other TSGs were also important, including *SLC5A8*.

## Supporting information

S1 Fig PROSPERO registration message. (DOC)

S2 Fig PRISMA 2009 checklist. The PRISMA (Preferred Reporting Items for Systematic Reviews and Meta_Analyses) Checklist that is a 27 checklist items pertain to the content of a systematic review and meta-analysis. Discussion.
(DOC)

S3 Fig Plos One checklist. A 19 checklist items pertain to the content of a systematic review and meta-analysis on genetic association studies.
(DOC)

S4 Fig Search syntax. The syntax and mesh terms that was used in this Meta-analysis.
(DOC)

S1 Tables Tables of selected studies. The final candidate studies which were chosen for Meta-analysis.
(DOC)
