# Supplementary material for: Meta-analysis of promoter methylation in eight tumor-suppressor genes and its association with the risk of thyroid cancer
Source: PLoS One. 2017 Sep 19;12(9):e0184892. doi: 10.1371/journal.pone.0184892 (PMC5605048; doi:10.1371/journal.pone.0184892)
Supplement: S4 Fig — The syntax and mesh terms that was used in this Meta-analysis. (DOC) [file pone.0184892.s004.doc]

1. SEARCH Syntax in PUBMED:

| **Syntax No** | **SYNTAX** | **Description** | **Records Number** |
| --- | --- | --- | --- |
| 1 | (((Thyroid Cancer[Title/Abstract] OR Thyroid Neoplasms[Title/Abstract]) AND Methylation[Title/Abstract]) OR Epigenetic[Title/Abstract]) AND ("2000/1/1"[PDAT] : "2016/10/1"[PDAT]) | All thyoid cancer and epigenetic related articles | 45962 |
| 2 | (Thyroid[Title/Abstract] AND Methyl[Title/Abstract]) AND ("200/1/1"[PDAT] : "2016/10/1"[PDAT]) | All thyoid cancer and methyl related articles | 686 |
| 3 | (((((("Thyroid cancer"[Title/Abstract] OR "Thyroid Neoplasm"[Title/Abstract]) OR Thyroid[Title/Abstract]) OR "Thyroid Carcinoma"[Title/Abstract]) OR "Thyroid Adenoma"[Title/Abstract]) AND Methylation[Title/Abstract]) OR "DNA methylation"[Title/Abstract]) AND ("2000/1/1"[PDAT] : "2016/10/1"[PDAT]) | All thyoid cancer and methyl related articles | 23326 |
| 4 | **(Thyroid[Title/Abstract] AND methylation[Title/Abstract]) AND ("2000/1/1"[PDAT] : "2016/10/1"[PDAT])** | **All thyroid abnormalities and methylation related articles** | 318 |
| 5 | Search (((("thyroid cancer"[Title/Abstract]) OR "thyroid neoplasm"[Title/Abstract]) OR "thyroid adenoma"[Title/Abstract]) AND "methyl") AND ("2000/1/1"[Date - Publication] : "2016/10/1"[Date - Publication]) |  | 22 |
| 6 | **((("Thyroid cancers"[Title/Abstract] OR "thyroid cancer"[Title/Abstract]) OR "thyroid/abnormalities"[Title/Abstract]) AND ("methylation"[MeSH Terms] OR "methylation"[All Fields])) AND ("2000/1/1"[PDAT] : "2016/10/1"[PDAT])** | **All thyroid abnormalities related articles** | 132 |
| 7 | Search **((thyroid[Title/Abstract]) AND tumor[Title/Abstract]) AND ("2000/1/1"[Date - Publication] : "2016/10/1"[Date - Publication])** | Thyroid cancer related articles | [10318](https://www.ncbi.nlm.nih.gov/pubmed/?cmd=HistorySearch&querykey=21) |
| 8 | **((((thyroid[Title/Abstract]) AND tumor[Title/Abstract]) AND ("2000/1/1"[Date - Publication] : "2016/10/1"[Date - Publication]))) AND methylation** | **All thyroid tumors and methylation related articles** | 165 |
| 9 | **(((((("thyroid cancer") OR "thyroid carcinoma") OR "thyroid adenoma") OR "thyroid neoplasm") AND ("2000/1/1"[Date - Publication] : "2016/10/1"[Date - Publication]))) AND methylation[Title/Abstract]** | **All thyroid neoplasm and methylation related articles** | 154 |
| 10 | (((thyroid[Title/Abstract]) AND cancer[Title/Abstract]) OR carcinoma[Title/Abstract]) AND ("2000/1/1"[Date - Publication] : "2016/10/1"[Date - Publication]) | All thyroid neoplasm and thyroid cancer related articles | 299128 |

**$: pt tag ([pt]) simillar to MeSH tag is applied in the MEDLINE subset of PubMed. Then, I use the syntax number9 and 4,5 for selecting the relevant clinical trials .**

1. SEARCH Syntax in SCOPUS:

| **Syntax No** | **SYNTAX** | **Description** | **Records Number** |
| --- | --- | --- | --- |
| 1 | **( TITLE-ABS-KEY ( "thyroid cancer" )  OR  TITLE-ABS-KEY ( "thyroid neoplasm" )  OR  TITLE-ABS-KEY ( "thyroid adenoma" )  AND  TITLE-ABS-KEY ( "methylation" ) )  AND  PUBYEAR  >  1999** | **All thyroid tumors and neoplasms and methylation related articles** | **280** |
| 2 | **ABS ( "thyroid cancer" )  OR  ABS ( "thyroid neoplasm" )  OR  ABS ( "thyroid adenoma" )  AND  ABS ( "methylation" ) )  AND  PUBYEAR  >  1999** | **All thyroid tumors and neoplasms and methylation related articles** | **124** |
| 3 | TITLE-ABS-KEY ( "thyroid" )  AND  PUBYEAR  >  1999 | All thyroid neoplasm and thyroid cancer related articles | 110,859 |
| 4 | ( TITLE-ABS-KEY ( "thyroid cancer" )  OR  TITLE-ABS-KEY ( "thyroid carcinoma" )  AND  TITLE-ABS-KEY ( methyl ) )  AND  PUBYEAR  >  1999 | All thyroid tumors and neoplasms and methyl group related articles | 239 |
| 5 | TITLE-ABS-KEY ( thyroid )  AND  TITLE-ABS-KEY ( methylation )  AND  PUBYEAR  >  1999 | All thyroid and methylation articles | 625 |
| 6 | ABS ( "thyroid cancer" )  OR  ABS ( "thyroid neoplasm" )  OR  ABS ( "thyroid adenoma" )  AND  ABS ( "methyl" ) )  AND  PUBYEAR  >  1999 | Methyl in thyroid cancer and neoplasms | 18 |

**I use the syntax number 1 for selecting the relevant clinical trials .**

1. **SEARCH Syntax in Web of S**cience (thomson Reuters):

| **Syntax No** | **SYNTAX** | **Description** | **Records Number** |
| --- | --- | --- | --- |
| 1 | **You searched for:** **TOPIC:** ("thyroid cancer") *OR* **TOPIC:** (" thyroid carcinoma") *AND* **TOPIC:**(methylation)  **Timespan:** 2000-2016. **Indexes:** SCI-EXPANDED, SSCI, CPCI-S, CPCI-SSH, ESCI. | All thyoid cancer methylated related articles | 14,145 |
| 2 | **TITLE:**("methyl") *AND* **TITLE:** ("thyroid cancer") *OR* **TITLE:** ("thyroid neoplasm") *OR* **TITLE:** ("thyroid adenoma") *OR* **TITLE:** ("thyroid carcinoma") *OR* **TITLE:** ("thyroid cancer")  **Timespan:** 2000-2016. **Indexes:** SCI-EXPANDED, SSCI, CPCI-S, CPCI-SSH, ESCI. | All thyoid cancer related articles | 14,313 |
| 3 | **TITLE:** ("thyroid cancer") *AND* **TITLE:** ("methylation")  **Timespan:** 2000-2016. **Indexes:** SCI-EXPANDED, SSCI, CPCI-S, CPCI-SSH, ESCI. | **All thyoid cancer and methyl related articles** | 23 |
| 4 | **TOPIC:** ("thyroid cancer") *AND* **TOPIC:** ("methylation")  **Timespan:** 2000-2016. **Indexes:** SCI-EXPANDED, SSCI, CPCI-S, CPCI-SSH, ESCI. | **All thyoid cancer and methyl related articles** | 162 |
| 5 | **TITLE:**("methyl") *AND* **TITLE:** ("thyroid carcinoma") *OR* **TITLE:** ("thyroid cancer") *OR* **TITLE:** ("thyroid neoplasm")  **Timespan:** 2000-2016. **Indexes:** SCI-EXPANDED, SSCI, CPCI-S, CPCI-SSH, ESCI. | All thyoid carcinoma and methyl group related articles | 7,364 |
| 6 | **TITLE:**("methylation") *AND* **TITLE:** ("thyroid")  **Timespan:** 2000-2016. **Indexes:** SCI-EXPANDED, SSCI, CPCI-S, CPCI-SSH, ESCI. | **All thyoid cancer and methyl related articles** | 76 |

**I use the syntax number 3, 4 , and 6 for selecting the relevant clinical trials .**
