# Supplementary material for: Meta-analysis of promoter methylation in eight tumor-suppressor genes and its association with the risk of thyroid cancer
Source: PLoS One. 2017 Sep 19;12(9):e0184892. doi: 10.1371/journal.pone.0184892 (PMC5605048; doi:10.1371/journal.pone.0184892)
Supplement: S1 Tables — The final candidate studies which were chosen for Meta-analysis. (DOC) [file pone.0184892.s005.doc]

| **Table 1**: Characteristics of 18 selected studies related to the RASSF1 Gene. | | | | | | | | | | |
| --- | --- | --- | --- | --- | --- | --- | --- | --- | --- | --- |
| **First Author** | **Year** | **Country** | **Type of Tissue** | **Total Number of Cases** | **Histology of cancer** | **Number of methylated cases** | **Type of Control** | **Number of Controls** | **Number of methylated Controls** | **Techniques for methylation analysis** |
| Mariana Brait | 2012 | USA | Fresh Frozen Tissue | 44 | PTC (n=27)  FTC (n=7)  HürthleCell (n=2)  MTC (n=8) | 38 | Normal Tissue | 15 | 13 | QMSP |
| Taylor C. Brown | 2014 | Sweden | Fresh Frozen Tissue | 43 | FTH (n = 23)  FTA (n = 10)  FTC (n = 10) | 21 | Normal Adjacent Tissue | 29 | 1 | Methyl Screen technology |
| Karolina Czarnecka | 2011 | Poland | Fresh Frozen Tissue | 45 | PTC classic ( n=27)  PTC follicular (n=15)  PTC tall-cell (n=3) | 13 | Macroscopically  unchanged thyroid tissue | 11 | 2 | MSP |
| M. O. Hoque, | 2006 | USA | Fresh Frozen Tissue | 85 | Benign & malignant tumors | 15 | Normal Adjacent Tissue | 15 | 2 | QMSP |
| PengHou, | 2008 | USA | FFPE | 101 | FTC (n=65)  ATC(n=36) | 43 | Benign nodules | 42 | 11 | QMSP |
| Kunstman, John W. | 2013 | USA | FFPE | 41 | PTC (n= 41) | 7 | Normal Tissue | 18 | 1 | Methyl Screen Technology |
| Migdalska-Sek | 2011 | Poland | Fresh Frozen Tissue | 11 | PTC (n=11) | 11 | Normal Adjacent Tissue | 11 | 10 | MSP |
| Mohammadi-asl, Javad | 2011 | IRAN | FFPE | 25 | Malignant PTC tumors (n=25) | 7 | Normal Tissue | 25 | 3 | COBRA |
| Nobuki Nakamura | 2005 | USA | Fresh Frozen Tissue | 89 | PTC (n=42),  FTC (n=4),  MTC (n=5)  ATC (n=12)  HTT (n=23)  FA (n=3) | 27 | Normal Adjacent Tissue | 38 | 1 | MSP |
| Angela Santoro | 2013 | Southern Italy | FFPE | 44 | PTC (n=32)  ATC (n=5)  HTC (n=3)  MTC (n=4) | 9 | Normal Tissue | 1 | 0 | MSP |
| UndragaSchagdarsurengin | 2006 | Germany | Fresh Frozen Tissue | 48 | PTC (n=13)  FTC(n=10)  UTC(n=9),  MTC (n=6)  FA(n=10) | 43 | Normal Tissue | 12 | 9 | MSP |
| UndragaSchagdarsurengin | 2002 | Germany | Fresh Frozen Tissue | 43 | PDTC (n=1)  MTC (n=5)  FTC(n=10),  UTC (n=9)  PTC (n=13) | 27 | Normal Tissue | 5 | 1 | MSP |
| Josena K. Stephen | 2011 | USA | FFPE | 13 | PTC (n=11), 2  FTC (n=2) | 10 | Normal Tissue | 8 | 6 | MS-MLPA Assay |
| Mingzhao Xing | 2004 | USA | Fresh Frozen Tissue | 51 | PTC (n=30)  FTC (n=12)  Adenoma (n=9) | 32 | Normal Tissue | 14 | 1 | Q-MSP |
| Bingfei Zhang, | 2014 | China | FNAB | 42 | PTC (n=42) | 26 | Normal Tissue | 26 | 4 | Q-MSP |
| JIA-JING LEE | 2008 | Sweden | Fresh Frozen Tissue | 21 | FTC (n=21) | 9 | Normal Adjacent Tissue | 21 | 3 | Bisulfite Pyrosequencing |
| HyeSook Min | 2009 | Korea | FFPE | 56 | INPTC (n=30)  FTN-INPTC (n=26) | 48 | Benign nodules | 35 | 22 | Q-MSP |
| Josena K. Stephen | 2015 | USA | FFPE | 53 | GD and FTC (n=53) | 45 | Normal Adjacent Tissue | 53 | 17 | Q-MSP |

MSP: Methylation Specific PCR, FFPE: Formaline Fixed Paraffin Embeded Tissue, QMSP: Quantitative Methylation Specific PCR, COBRA: Combined Bisulfite Restriction Analysis , FA: Follicular Adenoma ,FNAB :Fine needle aspiration biopsy, FVPTC : Follicular variant of papillary thyroid carcinoma , NH: Nodular Hyperplasia, FTN-INPTC: follicular patterned thyroid nodule with incomplete nuclear features of papillary thyroid carcinoma , GD : Graves Disease,WTC: Well-differentiated thyroid carcinoma, PTC: Papillary thyroid carcinoma, FTC :Follicular thyroid carcinoma, AT: Anaplastic thyroid carcinoma , FA: Follicular adenoma , UTC: Undifferentiated thyroid carcinoma, HN: Hyperplastic Nodules,Quantitative methylation-specific PCR: Q-MSP, Real Time Quantitative Methylation Specific PCR: RTQMSP, FFPE: Formalin Fixed Paraffin Embedded Tissue, Q-MSP:Quantitative Methylation Specific PCR

| **Table 2**: Characteristics of 12 selected studies related to the P16 Gene. | | | | | | | | | | |
| --- | --- | --- | --- | --- | --- | --- | --- | --- | --- | --- |
| **First Author** | **Year** | **Country** | **Type of Tissue** | **Total Number of Cases** | **Histology of cancer** | **Number of methylated cases** | **Type of Control** | **Number of Controls** | **Number of methylated Controls** | **Techniques for methylation analysis** |
| CarstenBoltze | 2003 | Germany | Fresh frozen | 77 | PTC (n=16)  FTC (n=18)  PDTC (n=12)  UTC (n=13) | 42 | Normal Tissue | 15 | 2 | QMSP |
| Mariana Brait | 2012 | USA | Fresh frozen | 44 | PTC (n=27)  FTC (n=7)  HürthleCell (n=2)  MTC (n=8) | 2 | Normal Tissue | 15 | 1 | QMSP |
| M. O. Hoque, | 2006 | USA | Fresh frozen | 20 | Benign & malignant tumors | 5 | Normal Tissue | 15 | 2 | QMSP |
| Peng Wang | 2013 | China | Fresh frozen | 74 | PTC (n=74) | 20 | Normal Tissue | 21 | 1 | MSP |
| CarstenBoltze | 2003 | Germany | Tissue | 139 | PTC (n=42)  FTC (n=38)  PDTC (n=25)  UTC(n=34) | 36 | Goiter | 65 | 8 | MSP |
| H. Chang | 2015 | Korea | FFPE | 164 | PTC (n=164) | 12 | BenignTissue | 77 | 3 | Methyl Light PCR |
| Monika Migdalska-Sęk | 2011 | Poland | Fresh frozen | 11 | PTC (n=11) | 11 | Normal Adjacent Tissue | 11 | 10 | MSP |
| JavadMohammadi-asl | 2011 | Iran | FFPE | 25 | Malignant PTC(n=25) | 10 | Benign nodules | 25 | 7 | COBRA |
| UndragaSchagdarsurengin, | 2006 | Germany | Fresh frozen | 48 | PTC (n=13)  FTC(n=10)  UTC(n=9),  MTC (n=6)  FA(n=10) | 13 | Goiter | 22 | 1 | MSP |
| UndragaSchagdarsurengin | 2002 | Germany | Fresh frozen | 43 | PDTC (n=1)  MTC (n=5)  FTC(n=10),  UTC (n=9)  PTC (n=13) | 36 | Goiter and Normal tissues | 5 | 1 | MSP |
| Alfred King Yin Lam | 2007 | Australia | Blood | 44 | PTC (n=44) | 18 | Normal Tissue | 9 | 0 | MSP |
| RossellaElisei | 1998 | USA | FFPE | 20 | PTC (n=12)  FTC(n=8) | 7 | Normal Adjacent Tissue | 20 | 6 | MSP |

PDTC: Poorly differentiated thyroid carcinomas, WTC: Well-differentiated thyroid carcinoma, PTC: Papillary thyroid carcinoma, FTC :Follicular thyroid carcinoma, AT: Anaplastic thyroid carcinoma , FA: Follicular adenoma , UTC: Undifferentiated thyroid carcinoma, HN: Hyperplastic Nodules,Quantitative methylation-specific PCR: Q-MSP, Real Time Quantitative Methylation Specific PCR: RTQMSP, FFPE: Formalin Fixed Paraffin Embedded Tissue,Q-MSP:Quantitative Methylation Specific PCR, COBRA: Combined Bisulfite Restriction Analysis

| **Table 3**: Characteristics of 9 selected studies related to the TSHR Gene. | | | | | | | | | | |
| --- | --- | --- | --- | --- | --- | --- | --- | --- | --- | --- |
| **First Author** | **Year** | **Country** | **Type of Tissue** | **Number of Cases** | **Histology of cancer** | **Number of methylated cases** | **Type of Control** | **Number of Controls** | **Number of methylated Controls** | **Techniques for methylation analysis** |
| Mariana Brait | 2015 | USA | Fresh frozen | 88 | HN (n=6), FA(n=12)  AN(n= 6), AH(n=1),  MNG(n=6)HA (n=13)  PTC(n=27)FTC(n= 7)MTC(n= 8) | 44 | Normal thyroid tissue | 15 | 9 | QMSP |
| Kyung Hee Han | 2009 | Korea | Fresh frozen | 60 | PTC(n=60) | 27 | Normal adjusted | 60 | 14 | Bisulfite genomic sequencing |
| M. O. Hoque | 2005 | Maryland | Fresh frozen | 100 | PTC (n=23)  FTC(n=10)  UTC(n=9),  MTC (n=5)  LT(n=6)  Adenomas(n=36) | 63 | Adjacent to benign nodules | 15 | 4 | Methyl Screen technology |
| KinyasKartal | 2015 | Turkey | FNA | 34 | PTC(n=15)  AUS-M(n=13)  FN-M(n=6) | 24 | Benign | 35 | 16 | MSP |
| Mosin S. Khan | 2014 | India | Fresh frozen | 60 | Thyroid tumor tissues (n=60) | 15 | Normal adjusted | 60 | 2 | MSP |
| JavadMohammadi-asl | 2011 | Iran | FFPE | 25 | Malignant PTC(n=25) | 11 | Benign | 25 | 7 | COBRA |
| UndragaSchagdarsurengin, | 2006 | Germany | Fresh frozen | 48 | PTC (n=13)  FTC(n=10)  UTC(n=9),  MTC (n=6)  FA(n=10) | 34 | Simple Goiter | 12 | 6 | MSP |
| Jason A. Smith, | 2007 | Little Rock | Fresh frozen | 32 | PTC (n=32) | 11 | Goiter+Benign | 27 | 2 | MSP |
| Mingzhao Xing | 2003 | USA | FFPE | 54 | PTC (n=39)  FTC (n=15) | 30 | Normal | 9 | 1 | MSP |

HN: Hyperplastic Nodules, MNG: Multinodular Goiters, HA: Hürthle Adenomas, LT: Cases with lymphocytic thyroiditis,AUS-M atypia of undetermined significance – Malignant, FN-M: Follicular carcinoma, WTC: Well-differentiated thyroid carcinoma, PTC: Papillary thyroid carcinoma, FTC :Follicular thyroid carcinoma, AT: Anaplastic thyroid carcinoma , FA: Follicular adenoma , UTC: Undifferentiated thyroid carcinoma, HN: Hyperplastic Nodules,Quantitative methylation-specific PCR: Q-MSP, Real Time Quantitative Methylation Specific PCR: RTQMSP, FFPE: Formalin Fixed Paraffin Embedded Tissue, Q-MSP:Quantitative Methylation Specific PCR, COBRA: Combined Bisulfite Restriction Analysis

| **Table 4**: Characteristics of 9 selected studies related to the SLC5AGenes. | | | | | | | | | | |
| --- | --- | --- | --- | --- | --- | --- | --- | --- | --- | --- |
| **First Author** | **Year** | **Country** | **Type of Tissue** | **Number of**  **Cases** | **Histology of cancer** | **Number of methylated cases** | **Type of Control** | **Number of Controls** | **Number of methylated Controls** | **Techniques for methylation analysis** |
| KatjaKiseljak-Vassiliades | 2011 | USA | Fresh Frozen | 138 | PTC(n=72)  Follicular variant PTC(n= 51)  tall-cell PTC(n= 8) | 45 | Normal tissues | 32 | 3 | MSP |
| Monika Migdalska-Sęk | 2011 | Poland | Fresh frozen | 11 | PTC(n=11) | 3 | Normal adjacent | 11 | 1 | MSP |
| Vale´riePorra | 2005 | France | Fresh frozen | 50 | PTC (n=31)  FTC(n=9)  FA (n=10) | 45 | Normal adjacent | 50 | 6 | MSP |
| Mingzhao Xing | 2003 | Maryland | Fresh frozen | 55 | PTC(n=35)  FTC(n=13)  ATC (n=7) | 36 | Normal adjacent | 9 | 4 | MSP |
| UndragaSchagdarsurengin, | 2006 | Germany | Fresh frozen | 48 | PTC (n=13)  FTC(n=10)  UTC(n=9),  MTC (n=6)  FA(n=10) | 10 | Goiter | 22 | 3 | MSP |
| Jason A. Smith | 2007 | USA | Tissue of microdissection | 32 | Malignant PTC(n=32) | 7 | Benign adjacenttissue | 27 | 0 | MSP |
| Josena K. Stephen | 2011 | USA | Tumoral Blocks | 16 | PTC (n=11)  FTC(n=2)  HT (n=3) | 9 | Normal Blocks | 5 | 1 | Methylation Specific Multiplex  Ligation-Dependent Probe Amplification (MS-MLPA) and MSP |
| Mariana Brait, | 2012 | USA | Fresh frozen | 44 | PTC (n=27)  FTC (n=7)  HürthleCell (n=2)  MTC (n=8) | 32 | Normal | 15 | 8 | QMSP |
| Ana LuizaGalrão, | 2013 | Brazil | - | 30 | PTC (n=18)  FTC (n=2)  Benign Tumors (n=10) | 20 | Non-tumoral surrounding  thyroid samples | 30 | 7 | semiquantitative-  MSP assay |

MS-MLPA: Methylation specific Multiplex Ligation-dependent Probe Amplification, WTC: Well-differentiated thyroid carcinoma, PTC: Papillary thyroid carcinoma, FTC :Follicular thyroid carcinoma, AT: Anaplastic thyroid carcinoma , FA: Follicular adenoma , UTC: Undifferentiated thyroid carcinoma, HN: Hyperplastic Nodules,Quantitative methylation-specific PCR: Q-MSP, Real Time Quantitative Methylation Specific PCR: RTQMSP, FFPE: Formalin Fixed Paraffin Embedded Tissue,Q-MSP:Quantitative Methylation Specific PCR

| **Table 5**: Characteristics of 6 selected studies related to the CDH1 Gene. | | | | | | | | | | |
| --- | --- | --- | --- | --- | --- | --- | --- | --- | --- | --- |
| **First Author** | **Year** | **Country** | **Type of Tissue** | **Number of Cases** | **Histology of cancer** | **Number of methylated cases** | **Type of Control** | **Number of Controls** | **Number of methylated Controls** | **Techniques for methylation analysis** |
| Monika Migdalska-Sęk | 2011 | Lodz | FFPE | 11 | PTC (n=11) | 10 | Normal adjacent tissues | 11 | 7 | MSP |
| Dan Wang | 2014 | China | FFPE | 120 | PTC (n=120) | 53 | Goiter | 23 | 2 | Pyrosequencing |
| Karolina Czarnecka | 2011 | Poland | FFPE | 45 | PTC (n=45) | 24 | Goiter | 23 | 5 | MSP |
| M. O. Hoque | 2006 | Maryland | FFPE | 85 | HN (n=20)  Adenomas: 24  PTC (n=23)  MTC (n=5)  LT" (n=6) | 17 | Normal tissues | 15 | 1 | MSP |
| Jason A. Smith | 2007 | USA | Fresh Frozen | 32 | PTC (n=32) | 18 | Normal adjacent tissues | 27 | 0 | MSP |
| Kirk Jensen, | 2010 | Ukraine | - | 66 | PTC (n=66) | 26 | Unknown | 22 | 1 | MSP |

LT: Lymphocytic Thyroiditis, WTC: Well-differentiated thyroid carcinoma, PTC: Papillary thyroid carcinoma, FTC :Follicular thyroid carcinoma, AT: Anaplastic thyroid carcinoma , FA: Follicular adenoma , UTC: Undifferentiated thyroid carcinoma, HN: Hyperplastic Nodules,Quantitative methylation-specific PCR: Q-MSP, FFPE: Formalin Fixed Paraffin Embedded Tissue,Q-MSP:Quantitative Methylation Specific PCR

| **Table 6**: Characteristics of 5 selected studies related to the DAPK Gene. | | | | | | | | | | |
| --- | --- | --- | --- | --- | --- | --- | --- | --- | --- | --- |
| **First Author** | **Year** | **Country** | **Type of Tissue** | **Number of Cases** | **Histology of cancer** | **Number of methylated cases** | **Type of Control** | **Number of Controls** | **Number of methylated Controls** | **Techniques for methylation analysis** |
| Shuiying Hu | 2006 | Maryland | Serum DNA | 66 | PTC (n=66) | 12 | Normal adjacent tissues | 22 | 1 | Real-time quantitative methylation- specific PCR, |
| UndragaSchagdarsurengin | 2006 | Germany | FFPE | 48 | PTC (n=13)  FTC(n=10)  UTC(n=9),  MTC (n=6)  FA(n=10) | 8 | Goiter | 22 | 15 | MSP |
| Dan Wang | 2014 | China | FFPE | 120 | PTC (n=120) | 105 | Goiter | 23 | 10 | Pyrosequrncing |
| Bingfei Zhang | 2014 | China | FFPE | 79 | PTC(n=79) | 60 | Goiter and benign nodule | 38 | 4 | pyrosequencing and Q-MSP |
| M. O. Hoque | 2006 | Maryland | FFPE | 85 | HN (n=20)  Adenomas(n= 24)  PTC (n=23)  MTC (n=5)  LT" (n=6) | 12 | Normal adjacent tissues | 15 | 2 | MSP |

Quantitative methylation-specific PCR: Q-MSP, Real Time Quantitative Methylation Specific PCR: RTQMSP, FFPE: Formalin Fixed Paraffin Embedded Tissue, WTC: Well-differentiated thyroid carcinoma, PTC: Papillary thyroid carcinoma, FTC :Follicular thyroid carcinoma, AT: Anaplastic thyroid carcinoma , FA: Follicular adenoma , UTC: Undifferentiated thyroid carcinoma, HN: Hyperplastic Nodules,Q-MSP:Quantitative Methylation Specific PCR

| **Table 7**: Characteristics of 6 selected studies related to the RARb2Gene. | | | | | | | | | | |
| --- | --- | --- | --- | --- | --- | --- | --- | --- | --- | --- |
| **First Author** | **Year** | **Country** | **Type of Tissue** | **Number of Cases** | **Histology of cancer** | **Number of methylated cases** | **Type of Control** | **Number of Controls** | **Number of methylated Controls** | **Techniques for methylation analysis** |
| M. O. Hoque | 2006 | Maryland | FFPE | 85 | HN (n=20)  Adenomas(n= 24)  PTC (n=23)  MTC (n=5)  LT" (n=6) | 10 | Normal tissues | 15 | 3 | MSP |
| Mohammadi-asl, J | 2011 | Iran | FFPE | 25 | PTC (n=25) | 13 | Benign cases | 25 | 8 | MSP |
| UndragaSchagdarsurengin, | 2006 | Germany. | FFPE | 48 | PTC (n=13)  FTC(n=10)  UTC(n=9),  MTC (n=6)  FA(n=10) | 5 | Goiter | 22 | 0 | MSP |
| Dan Wang | 2014 | China | FFPE | 120 | PTC (n=120) | 60 | Goiter | 23 | 10 | Pyrosequencing |
| Bingfei Zhang | 2014 | China | Fine needle Aspiration Biopsies | 79 | PTC (n=79) | 41 | Nodule | 38 | 13 | Q-MSP |
| Guillaume Gauchotte | 2013 | France | FFPE | 59 | PTC (n=36)  FTC (=25)  ATC (n=17)  FA (n=33) | 2 | Normal thyroid tissues | 9 | 1 | - |

WTC: Well-differentiated thyroid carcinoma, PTC: Papillary thyroid carcinoma, FTC :Follicular thyroid carcinoma, AT: Anaplastic thyroidcarcinoma , FA: Follicular adenoma , UTC: Undifferentiated thyroid carcinoma, HN: Hyperplastic Nodules,Quantitative methylation-specific PCR: Q-MSP, Real Time Quantitative Methylation Specific PCR: RTQMSP, FFPE: Formalin Fixed Paraffin Embedded Tissue., Q-MSP:Quantitative Methylation Specific PCR

| **Table 8**: Characteristics of 6 selected studies related to the PTENGene. | | | | | | | | | | |
| --- | --- | --- | --- | --- | --- | --- | --- | --- | --- | --- |
| **First Author** | **Year** | **Country** | **Type of Tissue** | **Number of Cases** | **Histology of cancer** | **Number of methylated cases** | **Type of Control** | **Number of Controls** | **Number of methylated Controls** | **Techniques for methylation analysis** |
| Francisco Alvarez-Nuñez | 2006 | Spain | Fresh Frozen | 59 | PTC (n=46)  TFA (n=6)  TFC (n=7) | 32 | Normal tissues | 32 | 3 | MSP |
| Taylor C. Brown, | 2014 | USA | Fresh Frozen | 10 | FTH (n=43) | 2 | Normal adjacent tissues | 11 | 1 | MethylScreen technology |
| H. Chang, | 2015 | Korea | FFPET | 164 | PTC (n=164) | 20 | Benign  lesions | 77 | 4 | MethyLight polymerase chain reaction (PCR) |
| PengHou, | 2008 | Maryland | FFPET | 101 | FTC(n=64)  ATC (n=36) | 45 | Benign tissue | 42 | 15 | QMSP |
| Michael G. White, | 2016 | Chicago | FFPET | 16 | PTC (n=16) | 15 | Normal adjacent tissues | 13 | 1 | - |
| UndragaSchagdarsurengin, | 2006 | Germany | Fresh frozen | 48 | PTC (n=13)  FTC(n=10)  UTC(n=9),  MTC (n=6)  FA(n=10) | 5 | Goiter | 8 | 4 | MSP |

WTC: Well-differentiated thyroid carcinoma, PTC: Papillary thyroid carcinoma, FTC :Follicular thyroid carcinoma, AT: Anaplastic thyroid carcinoma , FA: Follicular adenoma , UTC: Undifferentiated thyroid carcinoma, HN: Hyperplastic Nodules,Quantitative methylation-specific PCR: Q-MSP, Real Time Quantitative Methylation Specific PCR: RTQMSP, FFPE: Formalin Fixed Paraffin Embedded Tissue., Q-MSP:Quantitative Methylation Specific PCR, FTA: Thyroid follicular adenomas, FTC: Follicular thyroid cancer, TFC:Thyroid follicular carcinomas, FTH:Follicular thyroid hyperplasia
