# Supplementary material for: Meta-analysis of promoter methylation in eight tumor-suppressor genes and its association with the risk of thyroid cancer
Source: PLoS One. 2017 Sep 19;12(9):e0184892. doi: 10.1371/journal.pone.0184892 (PMC5605048; doi:10.1371/journal.pone.0184892)
Supplement: S1 Fig — (DOC) [file pone.0184892.s001.doc]

PROSPERO Registration message [33484]

Inbox

CRD-REGISTER

Feb 8 (2 days ago)

to me

Dear Dr Khatami

Thank you for submitting details of your systematic review Epigenetics of thyroid cancer to the PROSPERO register. We are pleased to confirm that the record has been published on the database.

Your registration number is: **CRD42016033484**

You are free to update the record at any time, all submitted changes will be displayed as the latest version with previous versions available to public view. Please also give brief details of the key changes in the Revision notes facility. You can log in to PROSPERO and access your records at http://www.crd.york.ac.uk/PROSPERO

An email reminder will be sent to you on the anticipated completion date, prompting you to update the record.

Comments and feedback on your experience of registering with PROSPERO are welcome at: crd-register@york.ac.uk

Best wishes for the successful completion of your review.

Yours sincerely

Jimmy Christie

PROSPERO Administrator

Centre for Reviews and Dissemination

University of York

York YO10 5DD

t: +44 (0) 1904 321040

f: +44 (0) 1904 321041

e: CRD-register@york.ac.uk

www.york.ac.uk/inst/crd

PROSPERO is funded by the National Institute for Health Research and produced by CRD, an academic department of the University of York.
